# Base-excision restriction enzymes: expanding the world of epigenetic immune systems

**DOI:** 10.1093/dnares/dsad009

**Published:** 2023-05-06

**Authors:** Kenji K Kojima, Ichizo Kobayashi

**Affiliations:** Department of Computational Biology and Medical Sciences, Graduate School of Frontier Sciences, University of Tokyo, Tokyo 108-8639, Japan; Institute of Medical Science, University of Tokyo, Tokyo 108-8639, Japan; Genetic Information Research Institute, Cupertino, CA 95014, USA; Department of Computational Biology and Medical Sciences, Graduate School of Frontier Sciences, University of Tokyo, Tokyo 108-8639, Japan; Institute of Medical Science, University of Tokyo, Tokyo 108-8639, Japan; Research Institute for Micro-Nano Technology, Hosei University, Koganei-shi, Tokyo 184-0003, Japan; Laboratory of Genome Informatics, National Institute for Basic Biology, Okazaki-shi, Aichi 444-8585, Japan

**Keywords:** restriction-modification, base excision, Helicobacter pylori, gastric cancer, DNA glycosylase

## Abstract

The restriction enzymes examined so far are phosphodiesterases, which cleave DNA strands by hydrolysing phosphodiester bonds. Based on the mobility of restriction-modification systems, recent studies have identified a family of restriction enzymes that excise a base in their recognition sequence to generate an abasic (AP) site unless the base is properly methylated. These restriction glycosylases also show intrinsic but uncoupled AP lyase activity at the AP site, generating an atypical strand break. Action of an AP endonuclease at the AP site may generate another atypical break, rejoining/repairing of which is difficult. This PabI family of restriction enzymes contain a novel fold (HALFPIPE) and show unusual properties, such as non-requirement of divalent cations for cleavage. These enzymes are present in *Helicobacteraceae*/*Campylobacteraceae* and in few hyperthermophilic archaeal species. In *Helicobacter* genomes, their recognition sites are strongly avoided, and the encoding genes are often inactivated by mutations or replacement, indicating that their expression is toxic for the cells. The discovery of restriction glycosylases generalizes the concept of restriction-modification systems to epigenetic immune systems, which may use any mode of damage to DNA that are considered ‘non-self’ based on epigenetic modifications. This concept will add to our understanding of immunity and epigenetics.

## 1. Introduction: restriction-modification systems

Most prokaryotes are now known to possess immune mechanisms that act at the genome/epigenome level. These include restriction-modification,^1^ CRISPR-Cas,^2^ Pgl/BREX,^3^ Argonaute,^4^ and abortive infection^5^ systems. Among them, restriction-modification systems directly depend on epigenetic modifications for distinguishing between ‘self’ and ‘non-self’.

**Figure 1.**
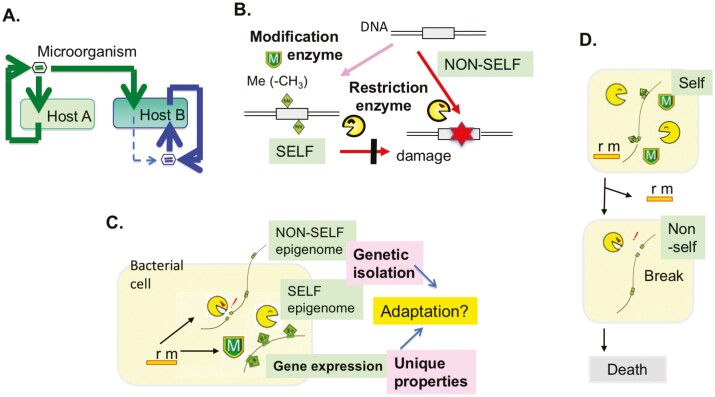
Restriction-modification systems as the prototype of epigenetic immune systems. (A) Discovery. Microbial adaptation to its host in the case of bacteriophage and bacteria. (B) Concept. ”Self” and ‘non-self’ DNA sequences are distinguished by the presence of an epigenetic modification such as base methylation. The non-self DNA is attacked by the restriction enzyme. Duplex lines, double-stranded DNA; Box, specific DNA sequence; Magenta diamond, epigenetic modification such as base methylation. (C) Plausible dual roles in adaptation. The lineage is isolated from the others. Methylation of a specific sequence at many sites along the genome generates a specific gene expression pattern and associated unique properties. (D) Autoimmune reaction (post-segregational killing or genetic addiction) by a Type II restriction-modification system. Loss of a restriction-modification system gene complex from a cell leads to decrease in genome methylation in the descendant cells. These genomes in the ‘non-self’ state will be attacked by the restriction enzyme to cell death.

The restriction-modification phenomenon was discovered in studies on microbial adaptation to the host (Fig. 1A). A bacteriophage grown in a host bacterium may not multiply well in another host, but its rare progeny can multiply well in this second host. This line appears to have learned how to grow in a single generation, which may be considered an example of inheritance of acquired traits, as proposed by Lamarck. Studies have shown that the underlying mechanism involves epigenetics,^1^ and that this represents a good model for studying transgenerational epigenetic inheritance.

In Type I, II, and III restriction-modification systems, a modification enzyme adds an epigenetic DNA modification that results in the DNA being recognized as self (Fig. 1B and C, Table 1). In a Type IV restriction system, DNA containing a specific epigenetic modification is regarded as non-self (Table 1).^6,7^ The modification distinguishing between self and non-self includes base methylation, its derivative modifications, and phosphorothioation.^8^ These four types of restriction-modification systems attack, by inducing breakage, foreign DNA sequences, such as phage DNA, which contain a specific epigenetic modification status and potentially provide defense against infection.

**Table 1.** Epigenetic immune systems and their candidates

Group	Subgroup	Epigenetic label (M)	Attack on genome (R)	Autoimmune action	Occurrence	Refs.
Restriction-modification	PabI	Base methylation	Glycosylase (base excision)	Post-segregational killing	Prokaryote	^9–11^
	Type IIP	Base methylation	Phosphodiesterase	Post-segregational killing	Prokaryote, Chlorella virus	^12,13^
	Type III	Base methylation	Phosphodiesterase		Prokaryote	
	Type I	Base methylation	Phosphodiesterase	Attack on chromosome (at replication fork?)	Prokaryote	^14^
	Type IV	Base methylation and its derivatives: hydroxymethylcytosine (5hmC) and its glucosyl derivative (5ghmC), phosphorothioation	Phosphodiesterase on modified DNA	Attack on chromosome (at replication fork?)	Prokaryote	^6–8^
DISARM		Base methylation	Phosphodiesterase		Prokaryote	^15^
BREX/Pgl	1,2,3,5,6	Base methylation	?		Prokaryote	^3^
BREX/Pgl	4	Phosphorothioation	?		Prokaryote	^3^
Dnd		Phosphorothioation (ds)	Phosphodiesterase		Prokaryote	^16^
Ssp		Phosphorothioation (ss)	Phosphodiesterase (ss break) by SspE		Bacteria	^17,18^
Dpd		7-Deazaguanine derivative	Phosphodiesterase		Bacteria	^19^
Uracil *N*-glycosylase		Base methylation (thymine as methylated uracil)	Glycosylase (base excision)	Thymineless death	Prokaryote, Eukaryote	^20^
TET/ Thymine DNA glycosylase		Cytosine without methylation	Thymine DNA glycosylase on Tet-modified m5C	Cell death?	All kingdoms, Phage	^21^

### 1.1. Autoimmune reactions by restriction-modification systems

Similar to that observed for other immune systems, restriction-modification systems exhibit autoimmunity. When a specific epigenetic modification system starts modifying the genome, a Type IV restriction enzyme attacks the genome and causes cell death.^22^ This acts as an effective defense against invasion by an epigenetic system in a structured habitat.^23^

In Type II restriction-modification systems, the role of the autoimmune process is evident in post-segregational killing, also known as genetic addiction, which involves the killing of bacterial cells that have lost the restriction-modification gene pair. When a bacterial cell loses a restriction-modification gene pair, its descendant cells lose chromosomal methylation and are killed by the remaining restriction enzyme molecules (Fig. 1D).^24^ Therefore, the restriction-modification system can be considered a type of toxin-antitoxin system.^25^ Post-segregational killing helps in maintaining a toxin-antitoxin system in structured habitats.^26^

Type I restriction enzymes attack host bacterial chromosome^14^ presumably at the DNA replication fork.^27^

### 1.2. Restriction-modification systems as mobile epigenetic elements

These findings on autoimmunity, or apparent conflicts between restriction-modification systems and their host ­bacteria, led to the hypothesis that restriction-modification systems are selfish mobile genetic elements such as transposons and viruses.^28^ This concept is supported by many lines of evidence from laboratory experiments and genome/epigenome analyses.

Restriction-modification systems show mobility and association with genome rearrangements, have sophisticated mechanisms regulating gene expression to ensure their maintenance, and are involved in interactions with other restriction-modification systems.^29^ In Type I and III restriction-modification systems, the unit of mobility can be as small as the target recognition domains that recognize methylation motifs.^30,31^ Some restriction-modification systems frequently switch their target sequences by replacing the target recognition domain.^32^ Competition for a recognition sequence within a bacterium^33^ may have led to individual specificity and collective diversity in recognition sequences. Theoretical studies have suggested that the presence of multiple restriction-modification systems of different specificities allows the coexistence of multiple bacterial lineages in the microbiome.^34^

### 1.3. Ever-changing hubs of a gene expression regulation network

Genome methylation by methyltransferases, either as components of restriction-modification systems or on their own (solitary methyltransferases), may affect gene expression and phenotype, depending on the methylation motif. Transcriptome and methylome analyses of the knockouts of many DNA methyltransferases in *H. pylori* demonstrated that they act as hubs in a gene expression regulation network, each controlling a specific set of adaptive phenotypes.^35^ The unique property of this network is that the hub methyltransferases frequently change their sequence specificity by replacing the target recognition domains (see above) and remodel the network. This frequent metamorphosis may lead to adaptive diversification.^31,32^ Post-segregational killing may enforce the action of this network on the genome by eliminating cells that are against the network.

## 2. An unusual restriction enzyme family

All the characterized restriction enzymes are phosphodiesterases that hydrolyse phosphodiester bonds in the DNA backbone. Recent studies have identified restriction enzymes that possess different activities.

### 2.1. Discovery based on gene mobility

**Figure 2.**
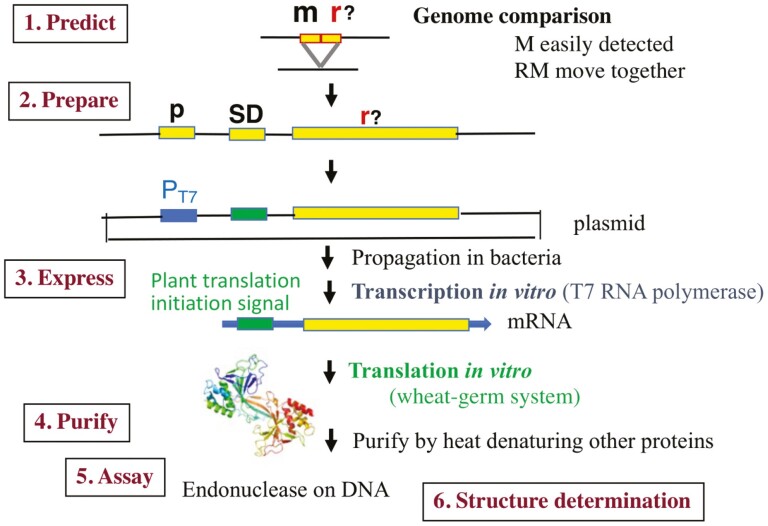
Search for a restriction enzyme with novel structure based on mobility of restriction-modification gene complex. 1. Closely related prokaryotic genomes were compared and the genes at a rearrangement joint nearby a DNA methyltransferase homolog were targeted. DNA methyltransferase genes are easily recognized by their amino-acid sequence motifs. 2. To bypass their potential toxicity to bacterial cells, each of these candidate genes (PCR-amplified or synthesized) was placed under P_T7_, a strong promoter, and a plant translation signal instead of a bacterial translation signal (Shine-Dalgarno sequence).^39^ The resulting plasmid was successfully propagated in *E. coli*. 3. They were subjected to *in vitro* transcription with RNA polymerase for the promoter and then for *in vitro* translation based on the plant (wheat germ) system. 4. Genes from thermophilic bacteria were initially tested because their products can be easily purified through heat denaturation of other proteins. 5. The product was assayed for endonuclease activity by adding dsDNA. 6. The structure of the protein prepared *in vitro* was determined using X-ray crystallography. The protein picture is by PyMOL (https://pymol.org) from PDB (ID 2dvy).

The divergence in the sequence and structure of restriction enzymes is limited.^1,36^ The most abundant superfamily is the PD-(D/E)xK superfamily. Other superfamilies include GIY-YIG endonucleases, HNH endonucleases, and PLD nucleases. To identify restriction enzymes with entirely novel structure and function, the mobility of restriction-modification genes, or more accurately, the co-mobility of a restriction enzyme gene and a modification enzyme (DNA methyltransferase) gene was used (Fig. 2).^9,37^ As DNA methyltransferase genes are readily recognized by the presence of characteristic amino acid motifs,^38^ closely related prokaryotic genomes were compared and the genes (i) that paired with a DNA methyltransferase gene and (ii) were at the genome rearrangement joint were listed. Among them, genes lacking sequence similarity to known restriction enzymes were targeted.

To bypass their potential toxicity to bacterial cells, each of these candidate proteins was expressed in *in vitro* transcription-translation system based on plant (wheat germ) translation.^9,39^ The products were assayed for restriction endonuclease activity in the dsDNA endonuclease-free extract. Genes in hyperthermophilic organisms were tested first, as their products can be easily purified *via* heat denaturation of other proteins.

A restriction enzyme, R.PabI (=PabI), and its paired methyltransferase, M.PabI, were identified from a hyperthermophilic archaeon, *Pyrococcus abyssi,* in this way. The gene pair appeared to have been inserted into the *P. abyssi* genome relatively recently considering its lower GC content and biased codon usage. M.PabI generates 5ʹ-GTm6AC from 5ʹ-GTAC.^40^ R.PabI appeared to cleave DNA at 5ʹ-GTA|C to generate TA-3ʹ overhang^9^ unless A is methylated to m6A.^40^ When dsDNA is hemimethylated, only the unmethylated strand is cleaved.^10^ Therefore, R.PabI and M.PabI form a Type II restriction-modification system. When expressed in *Escherichia coli,* R.PabI limited bacteriophage growth.^41^

R.PabI was found to be active at temperatures of up to 90°C. The hyperthermophilicity of M.PabI meant that it was active up to 95°C, which allowed the measurement of thermodynamic parameters for the first time in DNA methyltransferases.^40^

This research design is useful for detecting novel protein structures in mobile/horizontally transferred DNAs.^42^

### 2.2. Unusual properties

The sequence of R.PabI is not similar to that of any known restriction enzyme. In addition, R.PabI exhibits several unusual properties.

**Figure 3.**
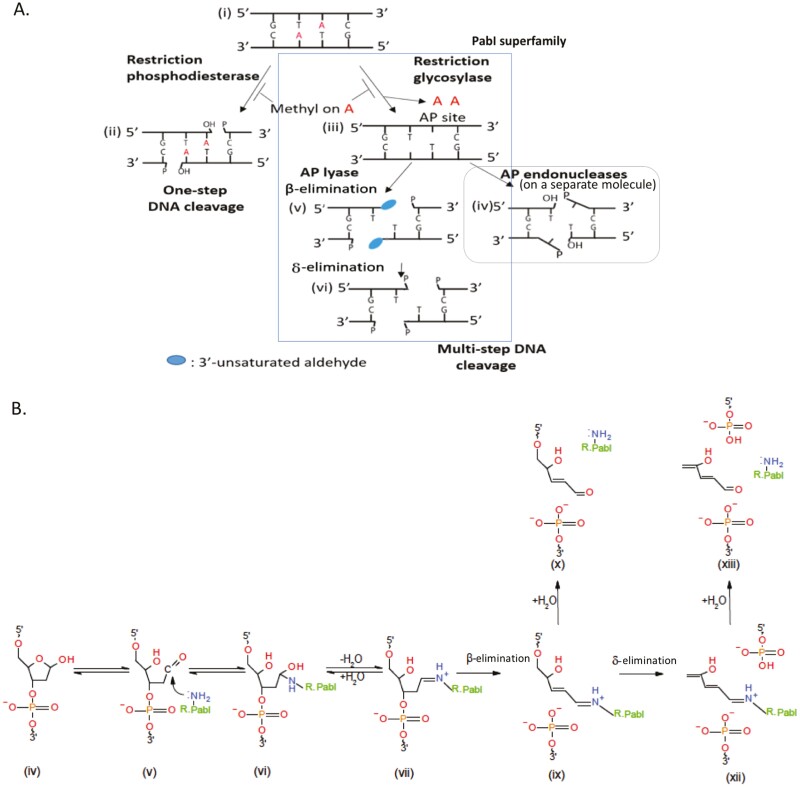
Reactions. (A) DNA cleavage pathways involving a typical Type II restriction endonuclease (restriction phosphodiesterase) and the PabI family of restriction glycosylases. (i) Double-stranded DNA with a restriction enzyme recognition sequence. (ii) Hydrolysis of phosphodiester bonds to generate breaks with 3ʹ-OH and 5ʹ-P ends (representing all the previously analysed restriction enzymes, albeit hypothetical). (iii) Base excision to generate abasic (AP) sites (PabI family). (v) AP lyase activity generates strand breaks with 3ʹ-modified sugar and 5ʹ-P ends, and (vi) breaks with 3ʹ-P and 5ʹ-P ends. Both pathways were inhibited by the methylation of a base (adenine, A in red) in the recognition sequence (5ʹ-GTAC). (iv) AP endonucleases on a separate molecule may act on the AP sites and generate breaks with different structures. Modified from^41^ (Oxford University Press, Creative Commons license). (B) Details of the reactions. Modified from^10^ (Oxford University Press, Creative Commons license). Roman numerals are discontinuous and unrelated to those in A.

(i) All previously known families of restriction enzymes hydrolyse the phosphodiester bond in the backbone of DNA and produce 3ʹ-OH and 5ʹ-phosphate ends (Fig. 3) that can be rejoined by DNA ligases. However, the ends generated by highly purified R.PabI cannot be re-ligated.^10,41^ The products showed a diffuse band in gel electrophoresis, suggesting heterogeneity in the end structure.^9^

(ii) All previously known families of restriction enzymes, with the exception of the PLD family, require a divalent cation for DNA cleavage. However, R.PabI does not require Mg^2+^ or other divalent metal ions.^11^

### 2.3. Homologs in Helicobacter and Campylobacter

**Figure 4.**
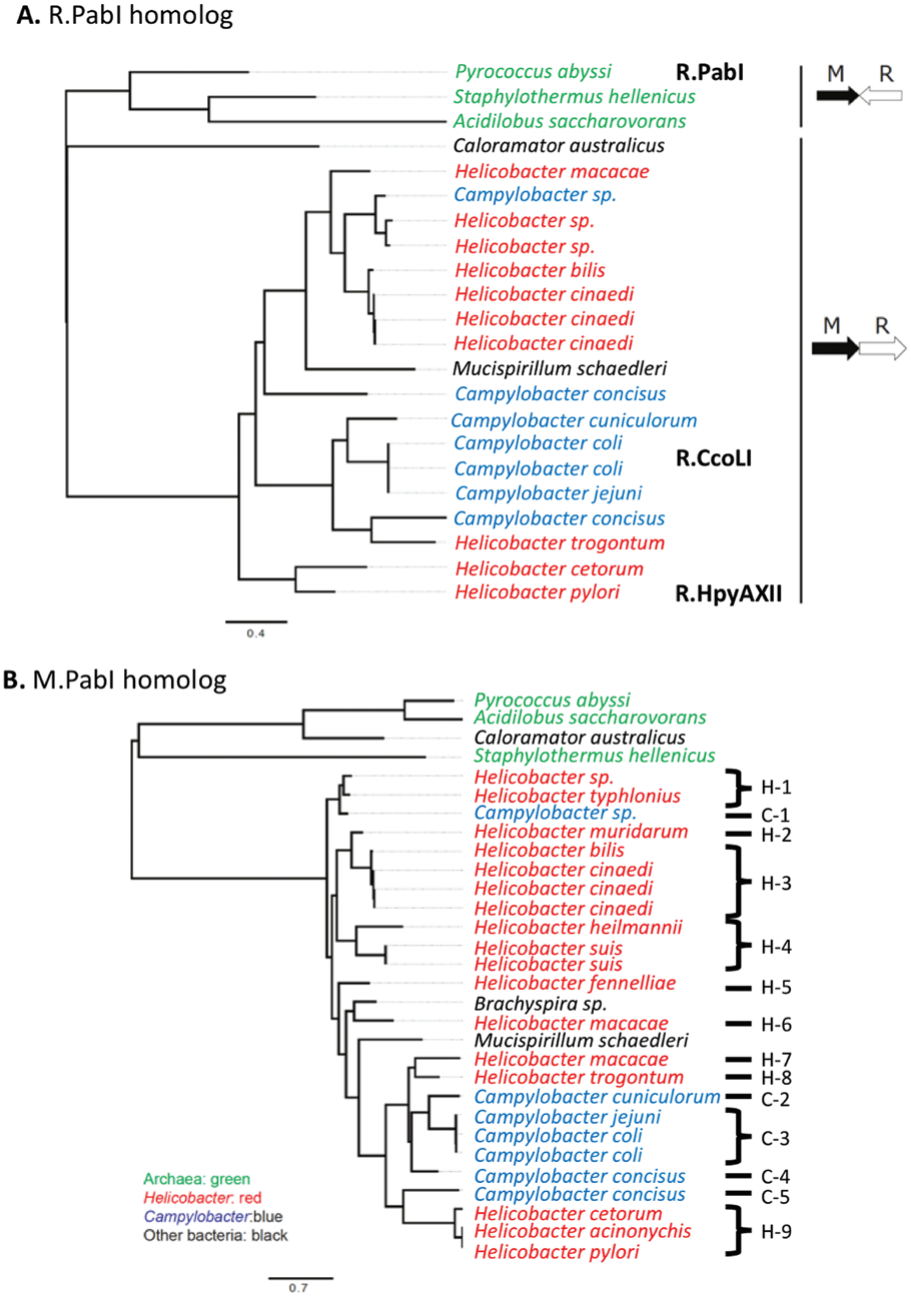
Distribution and phylogeny of PabI restriction-modification systems. Phylogenetic trees of R.PabI and M.PabI homologues. (A) R.PabI homologues. (B) M.PabI homologues. The orthologous loci are indicated. Modified from Fig. 1 in^43^ (Creative Commons License 4.0.).

R.PabI homologues are found in few thermophilic bacteria and archaea, and are abundant in the species of *Campylobacter* and *Helicobacter*, both of which belong to the order Campylobacterales (Fig. 4A).^9,43,44^ Homologous genes of R.PabI are always found next to a methyltransferase homolog gene, as expected for a restriction-modification system^11,43^ although their relative arrangements differ between archaea and *Helicobacter/Campylobacter* (Fig. 4A).

The *H. pylori* homologues of PabI were identified^45^ and named M.HpyAXII and R.HpyAXII (encoded by *hpyAXIIM* and *hpyAXIIR*, respectively)^46^ after the standard nomenclature of restriction-modification enzymes.^47^ We use these names for all *H. pylori* strains. Similar to M.PabI, M.HpyAXII was shown to methylate 5ʹ-GTAC to 5ʹ-GTmAC.^46^ In *E. coli* extract, R.HpyAXII was shown to target GTAC to generate double-strand breaks.^46^ Purified R.HpyAXII and R.CcoLI, a *Campylobacter coli* homolog, showed the same cleavage activity.^41^ R.CcoLI also showed single-strand cleavage activity on a closed circle plasmid and hemimethylated DNA.

In *H. pylori,* R.HpyAXII restricts the incoming plasmid and chromosomal DNA unless it is methylated by M.HpyAXII.^46^ It also affected the integration length.^48^ Similar to R.PabI, R.HpyAXII, and R.CcoLI expressed in *E. coli* limited phage growth in plaque assays.^41^ R.PabI and R.CcoLI also limited transformation by plasmids and attacked the endogenous chromosomes.

### 2.4. HALF PIPE fold

**Figure 5.**
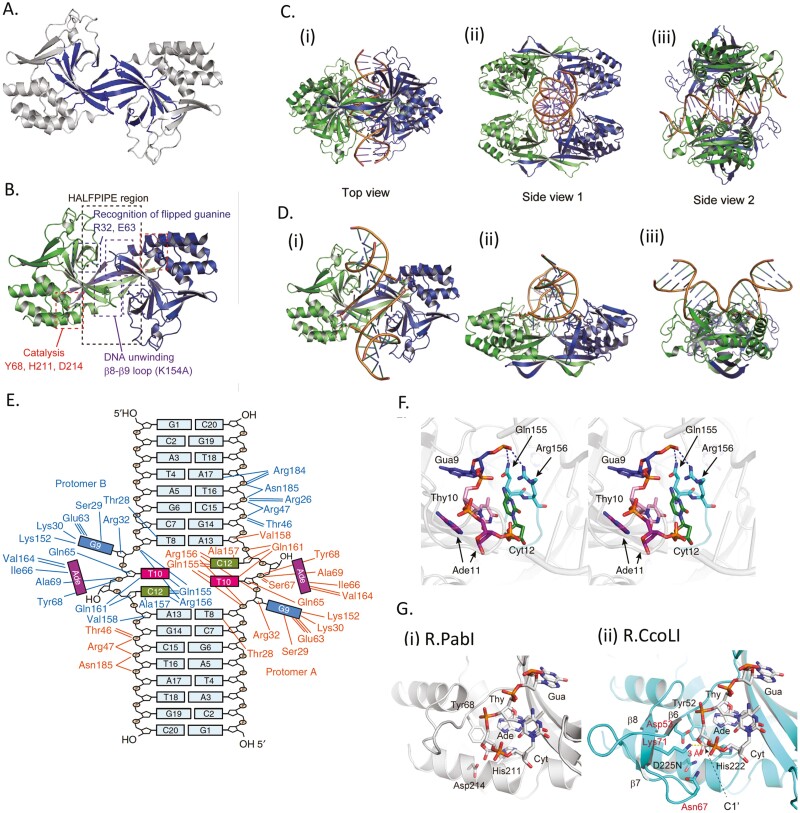
Structures. (A) R.PabI dimer (PDB ID: 2DVY). The half-pipe region is shown in blue. Reproduced from^11^ (Oxford University Press, Creative Commons license). (B) Important elements of R.PabI dimer (PDB ID: 3WAZ). Reproduced from^49^ (Oxford University Press, Creative Commons license). (C) Tetrameric structure of the complex of R.PabI with non-specific (without 5ʹ-GTAC-3’) dsDNA^49^ (Oxford University Press, Creative Commons license). R.PabI (R32A-E63A)-dsDNA complex (PDB ID: 5IFF). (D) R.PabI (K154A) bound to glycosylase products as in E (PDB ID 3WAZ). Reproduced from^49^ (Oxford University Press, Creative Commons license). The hydrolysed adenine site is shown as sticks. (E) Complex of R.PabI (K154A) with glycosylase products from DNA with a recognition sequence 5ʹ-GTAC-3ʹ. Inter-molecular hydrogen bonds are shown for each of the two R.PabI monomers. Reproduced from^11^ (Creative Commons BY license). (F) Stereo view of the glycosylase products, which included an AP site in 5ʹ-GT#C-3ʹ (# = abasic site), a free adenine, and R.PabI (K154A) (PDB ID 3WAZ). Reproduced from^11^ (Creative Commons BY license). Hydrogen bonds are indicated by blue dotted-lines. (G) Active sites for glycosylase and AP lyase. (i) R.PabI (K154A) binds to glycosylase products, DNA with the AP site (5ʹ-GT#C-3ʹ), and adenine. The three glycosylase active-site residues are shown using stick models and are labelled. Reproduced from^49^ (Oxford University Press, Creative Commons license). (ii) Model of R.CcoLI bound to glycosylase products. The three glycosylase active-site residues are shown using stick models and are labelled in black. The residues in characteristic β-sheet-containing regions are shown using stick models and labelled in red. Reproduced from^49^ (Oxford University Press, Creative Commons license).

Because of its toxicity to *E. coli* and presumably all forms of life, R.PabI was expressed in a wheat germ-based cell-free translation system on a large scale to prepare its crystal for X-ray diffraction^44^ (Fig. 2). This was the first report of a protein, the structure of which was solved using X-ray crystallography after preparation in a plant-based expression system. R.PabI adopts a novel protein fold. Homodimeric R.PabI, with rotational symmetry, has a curved anti-parallel beta sheet that forms a ‘half pipe’ (Fig. 5A) (PDB code:2DVY),^39,44^ which was named after the half pipe in the snowboard games in the Torino Olympic in 2006. Mutational and *in silico/in vitro* DNA-binding analyses have identified the positively charged groove as the dsDNA-binding site. R32, E63, and Y134 on/by the sheet (Fig. 5B) were found to be essential for cleavage, and R32 was involved in sequence-specific DNA binding.

Type II restriction enzymes move along dsDNA with sliding, hopping, and transfer movements in search of a recognition sequence.^12^ The crystal structure of R.PabI (R32A E63A double mutant) in complex with dsDNA lacking its recognition sequence was solved (PDB code:3WAZ).^49,50^ R.PabI forms a tetrameric structure, a dimer of the dimer, wrapping the dsDNA (Fig. 5C). The tetrameric structure was stabilized by four salt bridges between dimers at R70 and D71. Mutant analysis showed that these residues were essential for finding specific DNA sequences. However, these two residues are not conserved in *Helicobacter* or *Campylobacter* homologues. In R.PabI (Y68F K154A double mutant) in complex with a dsDNA lacking its recognition sequence, two R.PabI dimers interacted with one dsDNA. There was no contact between the dimers.^49^ In the co-crystal structure of R.PabI (Y68F K154A double mutant) and dsDNA containing the recognition sequence, R.PabI bent the DNA (PDB ID:6L2O),^49^ similar to that observed with some Type II restriction enzymes.^12^ The minor groove on the half pipe became wider than the major groove around the recognition sequence. A loop was inserted into the minor groove like a ‘wedge’. Between the two wedges, the base-stacking in one inter-base-pair interval was distorted. This structure is considered an intermediate of the cleavage reaction.

## 3. Base excision and AP lyase (endonuclease) activities

### 3.1. Base excision

In addition to the two unusual properties, difficulty in product end joining and non-requirement of divalent cations as discussed above, another peculiar feature was identified. After reaction with R.PabI at a low temperature (37°C), a plasmid remained supercoiled, which is free of strand breaks but without transformation ability.^10^ Prior methylation of adenine within the recognition sequence, generating 5ʹ-GTm6AC, prevented this loss of transformation ability. These observations indicated that surprisingly, restriction by PabI does not require DNA strand cleavage *in vitro*.

The co-crystal structure of R.PabI (K154A mutant, a weak cutter) and DNA with its recognition sequence revealed a novel reaction.^11^ The dimeric enzyme bent the duplex DNA at the recognition sequence by ~90° (Fig. 5D(iii)), unwound the duplex at 5ʹ-GTAC-3ʹ, and flipped out two nucleotides, G and A (Fig. 5E and F). Similar base flipping (nucleotide flipping) was found for another Type II restriction endonuclease family.^51^ Surprisingly, the adenine base was away from the backbone sugar (Fig. 5F). The DNA harboured two abasic sites (apurinic/apyrimidinic sites or AP sites, which have lost a base) on the two strands (Fig. 5E). This suggested that R.PabI catalyses the cleavage of the *N*-glycosidic bond of the adenine nucleotide as a DNA *N*-glycosylase during co-crystallization. dsDNA bending and base flipping are known functions of DNA *N*-glycosylases.^52^ Generation of AP sites was confirmed by treatment with DMED (*N,N*-dimethylethylenediamine), which specifically cleaves DNA strands at the AP sites.^10^ The generation of the free adenine base was detected using high-performance liquid chromatography, whereas the loss of adenine was verified by a decrease in the mass of the DNA using mass spectroscopy (MALDI-TOF MS).^11^

The AP sites generated by DNA glycosylases are unstable and cleaved easily. However, when the 5ʹ-GTAC-3ʹ-containing DNA duplex was treated with R.PabI at 40°C, cleavage was detected only after the addition of NaOH.^11^ Kinetic analysis and gel electrophoresis revealed that similar to other DNA glycosylases, R.PabI forms a tight complex with the product DNA.^10,11^ This base excision activity was shown to be specific for 5ʹ-GTAC-3ʹ in dsDNA and mismatched dsDNA.^11^ Base excision does not occur when the adenine is methylated,^10^ which shows that it forms a part of the restriction-modification system.

Three highly conserved residues, D214, H211, and Y68, are close to the adenine base in the excision products (Fig. 5B, E, and G(i)).^11^ D214A and D214N mutations reduced DNA binding and rendered the enzyme defective in base excision. D214 possibly provided a carboxylate ion for catalysis (Fig. 5G(i)). The Y68F mutant was defective in base excision, although DNA binding was not altered. Y68 was hypothesized to stabilize the catalytic water molecules. Mutant H211A showed reduced DNA binding, which is explained by its presence in the adenine-binding pocket, and reduced base excision, as explained by the presence of H211-Y68 hydrogen bonding. Characterisation of R.PabI as a DNA *N*-glycosylase led us to propose a new basic classification of restriction enzymes: restriction phosphodiesterases *versus* restriction glycosylases. Restriction phosphodiesterases include all known restriction enzyme families, other than the R.PabI superfamily (Table 1).

### 3.2. Intrinsic AP lyase (endonuclease)

What is the relationship between the base excision activity and DNA strand cleavage activity? Biochemical studies have demonstrated that R.PabI also possesses AP lyase activity, as it acts on the resulting AP site to generate a break with unusual end structures *via* β-elimination (Fig. 3A and B).^10^ The 5ʹ end is phosphorylated, while the 3ʹ end is a 3ʹ-phospho-α,β-unsaturated aldehyde. The δ-elimination reaction may follow. The AP lyase activity of R.PabI is not coupled with glycosylase activity, which explains the loss of transforming ability of a plasmid in the absence of strand breaks.^10^ This is not strictly sequence-specific, as R.PabI incised AP sites embedded in cognate (GT#C, # = AP site) and non-cognate (GC#C, AT#C, and GT#T, # = AP site) sequences with comparable efficiency.

Treatment of DNA carrying a single recognition site with R.PabI or its homologues, R.HpyAXII and R.CcoLI, in ­phosphate buffer yielded the same product *via* β-elimination.^41^ Another product was formed when NaOH was added, as expected, *via* δ elimination (Fig. 3). These results showed that the two homologues possess glycosylase and AP lyase activities. The AP lyase activity of R.CcoLI was confirmed using an oligonucleotide substrate with AP sites. How does the AP lyase reaction proceed? The AP lyase activity of DNA glycosylases requires an amino group, such as that in the lysine side chain. The amine group forms an imminium crosslink with C1’ of the deoxyribonucleotide (Fig. 3B(vi, vii)). The covalent ­DNA-enzyme reaction intermediate containing a Schiff base (Fig. 3B(vii)) was trapped by NaBH_4_ reduction in R.PabI^10^ and R.CcoLI.^41^

In a DNA docking model based on the crystal structure of R.CcoLI (D225N C189S mutant) (PDB ID:7CFA), conserved K71 (corresponding to K73 in R.PabI) (Fig. 5G(ii)) protrudes into the active site 3Å away from C1’ of the deoxyribose at the AP site.^53^ K71 appears to be stabilized by a β-sheet (β8), which is absent in R.PabI. As NaOH increases the formation of cleavage products with the R.CcoLI K71A D225N mutant compared to that with its D225N mutant, K71 was concluded to be important for AP lyase activity. K71A D225N generates a NaOH-cleavable product (AP site) faster than D225N. Thus, the action of K71 appears to be rate-limiting for glycosylases. This is consistent with the tight binding of R.PabI to the glycosylase product, as mentioned above. Direct measurement of AP lyase activity on defined substrates, such as oligo DNAs carrying AP sites, would further clarify these points. R.PabI might have relinquished its β-sheet (β8 in R.CcoLI) (Fig. 5G(i) vs. (ii)), which contributes to stronger AP lyase activity because AP sites are cleaved at high temperatures.^53^ As AP lyase is a form of endonuclease, the earlier claim that R.PabI is not a restriction endonuclease^11^ might be withdrawn.

These activities of DNA *N*-glycosylase and DNA AP lyase, leading to DNA cleavage, can explain the unique properties of strand breakage by R.PabI: non-requirement of divalent cations (see above), reluctance to re-ligation of the cleavage products (see above), and the diffuse electrophoretic mobility pattern of the cleaved DNAs.^9^

### 3.3. Extrinsic AP endonucleases versus repair

As many DNA glycosylases initiate base excision repair by AP endonuclease,^52^ we examined whether cellular DNA repair systems can alleviate the restriction mediated by the R.PabI family of DNA glycosylase/AP lyase. *E. coli* harbours two major AP endonucleases, endonuclease IV encoded by *nfo*^54^ and exonuclease III encoded by *xth*.^55^

Expression of R.PabI within *E. coli* causes restriction of incoming bacteriophages and endogenous chromosomal DNA (see above). Restriction at 37°C was suppressed by mutations in the genes encoding these two AP endonucleases.^41^ This indicates that, unexpectedly, AP endonucleases promote, but do not diminish, the restriction, presumably by introducing a DNA strand break (Fig. 3A). The enhancement of restriction by these AP endonucleases was also observed in the transformation of a break-free (closed circle) plasmid generated by reaction with R.PabI at 37°C *in vitro* (see above).^41^ Such effects were not observed with R. CcoLI. Treatment with R.PabI at 37°C, followed by treatment with endonuclease IV (an AP endonuclease), which is expected to result in OH-3ʹ and 5ʹ dRp ends (Fig. 3A), decreased transformation, which was not overcome by treatment with T4 ligase.

### 3.4. Difficulty in repairing unmethylated DNA

The above results suggest that the biological significance of the restriction glycosylases may lie in the difficulty in repairing the restricted products *via* end joining or template copying, especially unmethylated DNA. The dsDNA damage generated by restriction glycosylases resembles the damage generated by ionizing radiation or ‘radiomimetic’ antibiotics such as bleomycin.

In an attack on hemimethylated DNA, which is transiently generated during DNA replication/repair in cells carrying the PabI family restriction-modification system, an intact strand acts as a template for base excision repair of the damage. These are also generated during post-segregational killing. The chromosomal type II restriction-modification system HpyAXII resists its replacement by an empty site,^46^ likely *via* post-segregational killing.^56^ Indeed, chromosomal damage by these restriction glycosylases (R.PabI and R.CcoLI) is repaired by RecABC-mediated homologous recombination,^41^ similar to Type II restriction phosphodiesterases.^57^ Such ‘double standards’ toward ‘foreign enemy’ and ‘endogenous DNA’ have been suggested for Type I, II, and III restriction enzymes.^58–60^

The dsDNA breaks generated by restriction phosphodiesterases are repaired by homologous recombination mediated by phage function *via* the double-strand break repair model.^61^ Whether dsDNA breaks generated by restriction glycosylases can be repaired by double-strand break repair mechanisms is yet to be determined.

## 4. Toxicity suggested by evolutionary analyses

Based on our biochemical/biological analyses, we hypothesized that, compared to other restriction enzymes (restriction phosphodiesterases), the restriction glycosylases may more severely damage unmethylated foreign dsDNA, repair of which is difficult. We examined the family from an evolutionary/genomics point of view in search of evidence against this hypothesis of extra toxicity.

### 4.1. Exclusion with Ku for non-homologous end joining

**Figure 6.**
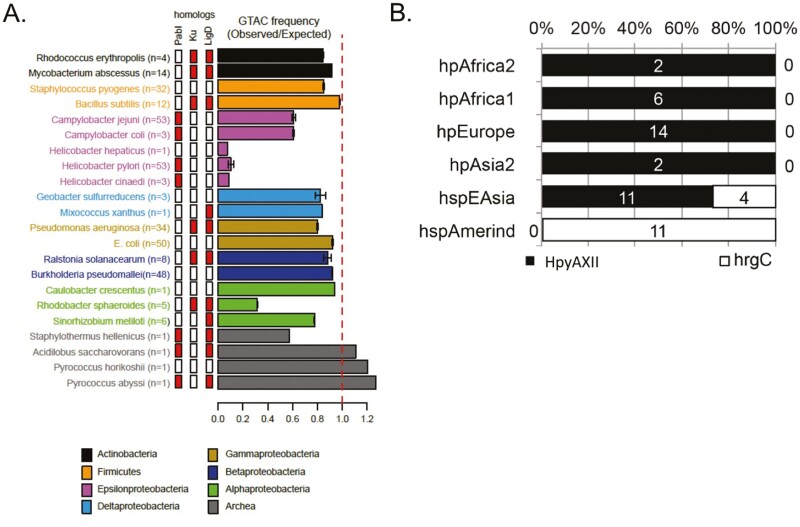
Evolutionary indications for the toxicity of PabI homologues. (A) Incompatibility with Ku-LigD and frequency of the recognition sequence. Species belonging to different taxa are shown in distinct colours. Bars indicate the mean and standard deviation. The central bars indicate presence (red) or absence (white) of a PabI family restriction-modification system, a Ku homolog, and a LigD homolog. n indicates the number of complete genomes analysed. From^41^ (Oxford University Press, Creative Commons license). (B) Replacement of HpyAXII by *hrgC* in multiple lineages in *H. pylori*. Distribution of the HpyAXII and *hrgC* in each genome population of *H. pylori*. From Fig. 4 in^43^ (Creative Commons License 4.0.).

When many genomes were compared, an apparent incompatibility was observed between the PabI and Ku homologues (Fig. 6A).^41^ Ku, a terminus-bridging protein with 5ʹ-dRP/AP lyase activity,^62^ is often accompanied by LigD, which also functions in DNA non-homologous end joining.^63^

Several hypotheses for their apparent incompatibility were proposed:

(i) The PabI homologues may have been ineffective because they cannot effectively damage the genome when Ku-LigD repairs dsDNA damage. This hypothesis is unlikely because Ku-LigD creates a deletion that is possibly detrimental to prokaryotic genomes.
(ii) Ku generates a deleterious deletion at the site of PabI-mediated dsDNA damage. Therefore, the presence of both systems is dangerous for the host. This can explain why PabI homologues are highly toxic. We have no evidence against this hypothesis now.
(iii) Ku-LigD homologues represent selfish elements that may introduce deleterious chromosomal deletions. Therefore, harbouring two selfish elements, PabI and Ku-LigD, will be too burdensome. This is not exclusive to hypothesis (ii). However, evidence regarding the selfish nature of the Ku-LigD is lacking.

### 4.2. Decrease in target sites (restriction avoidance) in the genome

The tetranucleotide, GTAC, recognized by the PabI family, is rarely found in *Campylobacter* and is even rarer in *Helicobacter* (Fig. 6A), as noted earlier (REBASE, http://tools.neb.com/~vincze/cutfreq/GTAC.html).^9,41,46^ This restriction avoidance is compatible with the long-term ­inheritance of the toxic PabI family members in *Helicobacter* and *Campylobacter*. This avoidance may have resulted from selective attack by the PabI family on resident or newly entering genomic DNA of the same group.

In contrast, GTAC avoidance was not observed in *Pyrococcus abyssi*.^64^ This could be due to the recent acquisition of the PabI family by *Pyrococcus*,^65^ the weaker AP lyase activity of R.PabI (see above), or the archaeal chromatin structure. The archaeal-type chromatin that evolved to eukaryotic chromatin was proposed to be an adaptation to restriction-modification systems.^64^

### 4.3. Long-term maintenance and mobility/genome rearrangements

In the *Helicobacter* and *Campylobacter* genomes, the genes encoding the R.PabI homolog and its partner methyltransferase were detected at 14 loci (Fig. 4),^43^ and they showed a cross-genus distribution in *Helicobacter* and *Campylobacter*. These results indicated the long-term maintenance of a gene pair with occasional horizontal transmission across an evolutionary timescale. Their mobility and associated genome rearrangements become evident when genomes of related strains/species in *Helicobacter/Campylobacter* are compared.^43^ The PabI homolog gene pair was inserted into operons for glutamate and histidine metabolism. As observed in the previously reported cases of operon insertion,^66^ restriction-modification systems may impose operon maintenance and expression in the host bacterium *via* post-segregational killing. A Type II restriction-modification system that recognizes GANTC was inserted into the *his* gene cluster, while its R gene was then replaced by the PabI homolog pair. These two restriction-modification systems with different restrictions and gene expression patterns appear to compete for a niche in this operon. Furthermore, there is evidence of competition within the PabI family. The genome of *Helicobacter macacae* harbours two distantly related PabI restriction-modification homologues, and one of the R genes is disrupted.

The PabI family is occasionally linked with other restriction-modification or toxin-antitoxin systems, as expected for ‘defense islands’.^67^ The *H. muridarum* genome harbours six M genes/pseudogenes and four R genes/pseudogenes between two non-restriction-modification genes.^43^ The PabI homolog appears to have been inserted with a 4-bp (TAAA) target site duplication and a 4-bp (palindrome AGCT) target deletion.

In summary, these PabI homologues appear to be active mobile elements.

### 4.4. Decay of R gene

The M.HpyAXII is highly conserved among *H. pylori* strains, whereas the R.HpyAXII is poorly conserved among *H. pylori* strains.^43,46^ PabI homologues have been inactivated by frameshift mutations, nonsense mutations, deletion mutations, and/or insertion of an integrative conjugative element. In a panel of clinical isolates of *H. pylori*, R.HpyAXII was ­functional in ~20% cases, whereas the activity of the M.HpyAXII was highly conserved (~90%).^46^ The decay of R.HpyAXII may have resulted from its toxicity. The solitary M.PabI homolog possibly exerts a vaccine-like effect on the PabI restriction-modification system, as has been shown for another system.^68^ The restriction-modification system may have been ­maintained for a long time by the three-component cycle of ‘virulent pathogen => costly vaccine => none => virulent pathogen’ as has been demonstrated theoretically.^69^

### 4.5. Replacement by a non-homologous gene

In many *H. pylori* strains lacking the HpyAXII restriction-modification system, most of the restriction and modification coding regions were substituted by a gene in reverse orientation, named *hrgC* (HpyAXII replacing gene C).^46^ HrgC homologues have been identified in various bacteria including *Escherichia coli* and *Bacillus cereus*. HrgC (HPF30_0819) was predicted using AlphaFold to contain transmembrane helices and an anti-parallel beta sheet (UniProt, https://www.uniprot.org/uniprotkb/E6NJG3/entry).

The distribution of the HpyAXII gene pair in global strains of *H. pylori* reflects the phylogeographic diversification associated with human migration. Replacement by *hrgC* occurred in two sub-populations of *H. pylori*, hspAmerind (hspIndigenousAmerica^70^) and hspEAsia, both of which were generated during the eastward movement of *H. pylori* (Fig. 6B). Subsequently, *H. pylori* with and without *hrgC* were intermixed. The lower contribution of hspAmerind to *H. pylori* genomes in the Americas than those of African and European lineages^71^ may be explained by the advantages of the HpyAXII restriction-modification system over HrgC.

### 4.6. Effects on gene expression and microcin synthesis

M.HpyAXII in *H. pylori* affects expression of the sequence-specific subunit of a Type I restriction-modification system.^41^ This is an example of hierarchical control between hub restriction modification systems in epigenetic gene regulation network.^35^ It also affected the expression of membrane proteins.

Unexpectedly, methyltransferase also stimulates the expression of microcin C7 biosynthesis genes, *mccA* and *mccB*, in a region with dense GTAC sites (the recognition sequence of HpyAXII) on a plasmid.^41^ Microcin C7 is an oligopeptide-nucleotide antibiotic.^72^ Production of microcin C in other bacteria switches when cells reach the stationary growth phase under regulation at the transcriptional level by growth phase regulators and global bacterial regulators.^73^ Transcription of this *mcc* operon in *H. pylori* is also affected by a methyltransferase targeting CATG, which overlaps with the start codon (ATG) of the first gene, *mccA*, of this microcin operon.^35^ The biological significance of these observations remains unclear, although this antibiotic operon may utilize the gene regulation network involving HpyAXII.

## 5. Further hypotheses regarding biological role of PabI

The above lines of evidence are consistent with the toxic effect of the PabI family of restriction glycosylases, especially on unmethylated DNA, as opposed to that on hemimethylated DNA. However, the target of this toxicity is not clearly understood. To understand this, we discuss the cases of *Helicobacter* and *Campylobacter*.

### 5.1. Attacking other bacterial genomes?

Instead of the incoming unmethylated DNAs, the toxicity of this system might be intended for targets outside the R.PabI-producing cells. Bacteria, including *Helicobacter* and *Campylobacter,* produce toxins and bacteriocins (such as microcin C7, mentioned above) to damage other bacteria in the microbiome.^74^ Similar to DNase bacteriocins, the restriction glycosylases themselves might leave the producing cells, invade other bacterial cells, and attack their unmethylated genomes.

How can restriction glycosylases achieve these goals? Linked genes are responsible for the secretion of bacteriocins, which take advantage of specific cellular uptake machinery, such as importers, to enter recipient cells. The R.PabI family lacks linked secretion genes and such a special structural feature.^43^ However, their DNA-binding ability may serve both functions. The R.PabI family can tightly bind dsDNA and might utilize DNA transfer machinery, such as conjugation machines or outer membrane vesicles,^75^ for transfer to other bacteria.

### 5.2. Attacking host genome (and causing cancer)?

Several bacterial ‘genotoxins’ target host DNA.^76^ These include typhoid toxins produced by *Salmonella enterica* serovar Typhi, cytolethal distending toxins produced by *Campylobacter* and *Helicobacter*, oncogenic Colibactin of *E. coli,* and histone methyltransferase of *M. tuberculosis.*^77^ Infection of human cells with *H. pylori* induces double-strand breakage in chromosomal DNA^78^ and expression of APE1 (an AP endonuclease).^79^ DsDNA breakage is only partially dependent on *cagPAI* and *cagA*.^80,81^ The CagPAI Type IV secretion system of *H. pylori* transfers DNA to host human cells.^82^ Using these DNA molecules, R.HpyAXII might be able to move into human cells, similar to *Agrobacterium* proteins, which move into plant cells *via* the Type IV secretion system.^83^ Outer membrane vesicles with dsDNA may also provide a route to human cells.

**Figure 7.**
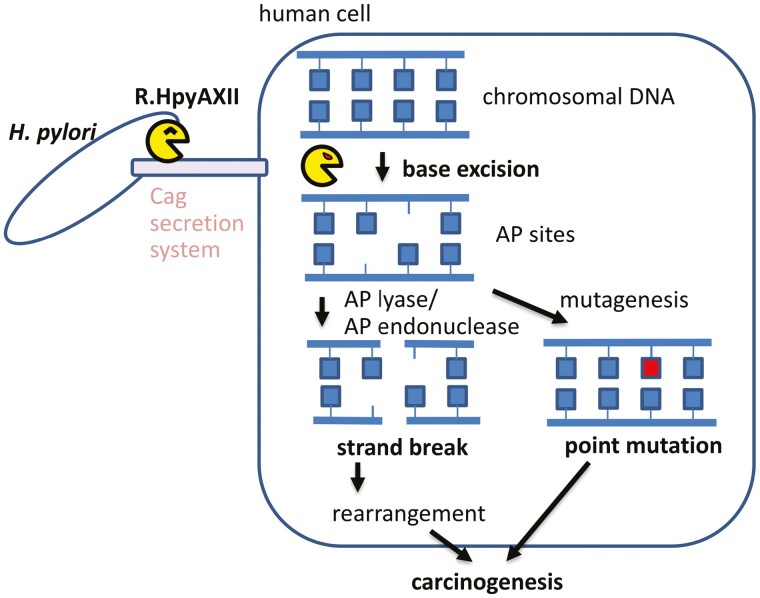
A hypothesis for *H. pylori’s* base-excision restriction enzyme and stomach cancer. *H. pylori* transfers R.HpyAXII restriction glycosylase bound to dsDNA to human cells *via* CagPAI Type IV secretion machinery. The glycosylase excises adenine base at 5ʹ-GTAC-3ʹ from the human chromosome. The action of AP lyase or human AP endonuclease on the resulting AP site generates double-strand breaks, which in turn leads to genome rearrangements and chromosome instability. The action of R.HpyAXII also leads to substitution mutations at A of 5ʹ-GTAC-3ʹ. These lead to stomach (gastric) cancer.

Colibactin from *E. coli* causes DNA damage and tumorigenesis^84^. *H. pylori* is responsible for most incidences of stomach cancer, and it induces mutations *via* unknown mechanisms.^85^ AP sites are known to be mutagenic,^86^ and the aberrant ends generated by AP lyase/AP endonuclease on the double AP sites on unmethylated human DNA may result in deletion and ­genome rearrangements. APE1 is known to process the ends of non-homologous end joining.^87^ The DNA glycosylase and AP lyase activities of R.HpyAXII and the endonuclease activity of APE1 in the human genome might cause mutations and genome rearrangements, leading to carcinogenesis (Fig. 7), similar to that observed with Colibactin and *Agrobacterium* proteins.^83,88^

## 6. Prospect: epigenetic immune systems

### 6.1. Related base excision reactions

Base excision has been extensively studied in the context of repair of damaged bases. However, several studies have demonstrated that base excision can damage viral and cellular genomes, stimulate recombination, and lead to chromosome replication arrest and cell death.^89^ One such enzyme, the human homolog of MutY DNA glycosylase, generates single-strand breaks and triggers cell death.^90^ The elimination of cells with damaged genome may help in the survival of a cell population.

The restriction glycosylase and active DNA demethylation systems are similar.^91^ In plants, demethylation of m5C occurs *via* direct excision of the methylated base by DNA glycosylases (called ROS1 and Demeter).^92^ In animal cells, by ten-eleven translocation dioxygenase, m5C is first converted to hydroxymethylcytosine (5hmC), which is further oxygenated and then excised by thymine DNA glycosylases.^93^

### 6.2. Uracil N-glycosylases

Base excision has been well studied for uracil *N*-glycosylases (UNGs), which excise uracil from DNA. They restrict the propagation of bacteriophages harbouring uracil instead of thymine in their genomic DNA.^20^ This process is similar to restriction by restriction enzymes, as uracil is an unmethylated form of thymine, although the methylation is genetic rather than epigenetic. This process may reflect ancient conflicts between uracil-based DNA (U-DNA) and thymine-based DNA (T-DNA) in the transition from the RNA world to the DNA world. Thus, U-DNA may survive in the form of phages.^94^ Thymine may have helped avoid this restriction. This process is analogous to the present arms race between restriction-modification and phages. Here, UNGs are analogous to a restriction glycosylase, and the genes in the thymine synthesis pathway correspond to its partner modification enzyme.

UNGs also act as eukaryotic defense systems against foreign DNA.^95^ Proteins of the APOBEC/AID family are cytosine deaminases that generate uracil in foreign DNA. UNGs generate a recombinogenic break in DNA with uracils,^96^ which is reminiscent of stimulation of recombination by Type II restriction endonucleases (phosphodiesterases).^61,97^

‘Thymineless death’, which refers to the suicide of cells that cannot synthesize thymine and thus incorporate uracil in place of thymine into DNA,^98^ is similar to restriction-mediated killing of cells that have lost the methyltransferase and may correspond to the autoimmune response of this system.

Some phages contain modified bases, such as hydroxymethyluracil (5hmU) instead of thymine, and 5hmC instead of cytosine, to avoid some restriction-modification systems.^6^ Some *Campylobacter* phages harbour I (inosine) instead of G on DNA.^99^ This may be comparable to the above roles of T/U in DNA. We speculate that inosine *N*-glycosylase might be present in *Campylobacter.* These arguments indicate that the current DNA world consisting of four bases (A, T, G, C) is a subspace of the epigenetic DNA world and not the other way round.

### 6.3. Generalization of restriction-modification systems

These considerations led us to generalize the concept of restriction-modification systems to ‘epigenetic immune/self-recognizing systems’ (Table 1). These consist of any type of DNA damage or block to DNA replication, which we define as generalized R, and any type of epigenetic DNA modification, which we define as generalized M. Recent studies regarding prokaryotic defense systems have helped us toward this end.^3,15,16,19,100–105^ Epigenetic modification is not restricted to base methylation, but also includes DNA backbone phosphorothioation, as reported for the *Dnd* system and *Ssp* system.^16–18^ Various complex modifications involving oxidation, glycosylation, and others have been suggested to be present in DNA using bioinformatics analysis^100^ and have been detected in phage genomes.^21,99,106–108^ One of them has been revealed to be a part of the defense system; the *Dpd* system, which modifies DNA with 7-deazaguanine derivatives, shows restriction of plasmid propagation.^19^ It seems not a coincidence that *Campylobacter* firehammervirus DNA shows complete replacement of deoxyguanosine with 7-deazaguanine derivatives.^99^ Given the frequent observation of host-phage arms-race, other types of DNA modifications seen in phage genomes are also likely incorporated in the prokaryotic immune system.

Generalized R can damage DNA or specifically interact with DNA-binding proteins, unless marked by generalized M. In addition to cleavage and base excision, what other type of DNA damage is sufficient to function as generalized R? One candidate for generalized R action is DNA ADP-ribosylation, which modifies phage DNA and prevents replication in the DarTG toxin-antitoxin system.^101^ Deamination is another candidate for generalized R action, considering the function of APOBEC3 in eukaryotes. Deaminase converts cytosine to uracil, which is then excised using UNG. Considering that UNG is a universal protein, C-to-U editing might be sufficient for preventing DNA replication.

A Type 1 bacteriophage exclusion (BREX) system in *Bacillus subtilis* inhibits the reproduction of unmethylated phage genomes without cleavage.^3^ In contrast, the PglX methyltransferase of the phage growth limitation (Pgl) system (a Type 2 BREX system) methylates phage DNA in *Streptomyces coelicolor*, and a Type IV restriction enzyme encoded at a distant locus of the genome cleaves the methylated phage DNA.^102^ In the presence of a Type IV restriction system, M can become R. The same reaction can ­fulfil the roles of R and M, depending on the context. PglX methyltransferase functions as a toxin, and PglZ phosphatase as an antitoxin. In addition to putative DNA-modifying enzymes, such as methyltransferases or phosphoadenylyl-sulfate reductases, Pgl/BREX systems encode different types of enzymes, such as helicases, proteases, kinases, phosphatases, and ATPases. Various mechanisms may block phage replication. The defense island system associated with restriction modification (DISARM) also contains either adenine methylase (*drmMI*) or cytosine methylase (*drmMII*).^15^ It is proposed that unmodified 5ʹ-DNA overhangs activate the DISARM system.^103^ DrmC, which contains a PLD nuclease, can act as an effector nuclease for phage degradation. Among the defense systems predicted based on ­bioinformatic survey,^104,105^ Druantia type II and Hma contain a methyltransferase as their component. The Hma system is composed of three genes, encoding methyltransferase, helicase, and ATPase, whereas the Druantia system type II is composed of four genes encoding methyltransferase, helicase, and two hypothetical proteins.

How are epigenetic and damaging modifications distinguished? 6-methyladenine, a typical epigenetic form of adenine in prokaryotes, is a damaged base in yeast.^109^ Uracil, which is excised by uracil *N*-glycosylases, can be considered either a damaged (deaminated) cytosine or an epigenetically modified (demethylated) thymine.

These generalized self-recognizing epigenetic systems are similar to bacterial toxin-antitoxin systems.^25^ Our analysis of prokaryotic immune systems suggests that they perform surveillance against non-self states and do not simply attack invading elements. The essence of immunity is autoimmunity, which is also a concept emerging from current mammalian immunology.

## 7. Conclusion

The discovery of restriction glycosylases, restriction enzymes that excise unmethylated bases from target DNA, led us to propose the concept of generalized epigenetic immune systems. They distinguish non-self states from self states using epigenetic signals and may play important roles in epigenetic processes in prokaryotes and eukaryotes. Their important role is in autoimmunity, that is watching themselves against ‘non-self’ states.

## Conflict of interests statement

IK is receiving a research grant from Synplogen Co.
